# Headache Is Associated with Low Systolic Blood Pressure and Psychosocial Problems in German Adolescents: Results from the Population-Based German KiGGS Study

**DOI:** 10.3390/jcm10071492

**Published:** 2021-04-03

**Authors:** Melissa A. Centeno Córdova, Daniela Stausberg, Biyao Wang, Andreas Becker, Aribert Rothenberger, Christoph Herrmann-Lingen, Thomas Meyer, Julia Staab

**Affiliations:** 1 Clinic for Psychosomatic Medicine and Psychotherapy, University Medical Center Göttingen (UMG), Waldweg 33, D-37075 Göttingen, Germany; melyctn@gmail.com (M.A.C.C.); d.stausberg@stud.uni-goettingen.de (D.S.); cherrma@gwdg.de (C.H.-L.); julia.staab@med.uni-goettingen.de (J.S.); 2 Clinic for Child and Adolescent Psychiatry and Psychotherapy, University Medical Center Göttingen (UMG), von-Siebold. Str. 5, D-37075 Göttingen, Germany; biyao.wang@med.uni-goettingen.de (B.W.); abecker4@gwdg.de (A.B.); arothen@gwdg.de (A.R.); 3 DZHK (German Centre for Cardiovascular Research), Partner Site Göttingen, 10785 Berlin, Germany

**Keywords:** headache, blood pressure, magnesium, quality of life, distress, children, adolescents

## Abstract

Studies have reported controversial results on the relationship between headache and blood pressure. The aim of this post hoc study was twofold: first, to further investigate this relationship and, second, to assess the impact of psychosocial factors on this association in a population-based study of German children and adolescents. The analysis was conducted on study participants aged between 11 and 17 years (*n* = 5221, weighted from the total study cohort) from the nationwide German Health Interview and Examination Survey for Children and Adolescents (KiGGS). Health-related quality of life was assessed by self- and parent-rated German-language KINDL-R questionnaires (Children’s Quality of Life Questionnaire), while mental problems were analyzed using the Strengths and Difficulties Questionnaire (SDQ). Our findings confirmed that blood pressure was significantly lower in adolescents reporting episodes of headache than in those without headache (114.0 ± 10.2 mmHg vs. 115.5 ± 11.0 mmHg, *p* < 0.001). Logistic regression models adjusted to sex, age, body mass index, contraceptive use, and serum magnesium concentration demonstrated that headache was significantly associated with self-rated KINDL-R (Exp(B) = 0.96, 95% confidence interval (95% Cl) = 0.96–0.97, *p* < 0.001), parent-rated KINDL-R (Exp(B) = 0.97, 95% CI = 0.96–0.98, *p* < 0.001), as well as self-rated SDQ (Exp(B) = 1.08, 95% CI = 1.07–1.10, *p* < 0.001), and parent-rated SDQ (Exp(B) = 1.05, 95% CI = 1.04–1.06, *p* < 0.001). There was evidence that quality of life and mental problems mediated the effect of blood pressure on headache, as revealed by mediation models. Our results from the nationwide, representative KiGGS survey showed that low blood pressure is a significant predictor of headache, independent of quality of life and mental problems. However, these psychosocial factors may mediate the effect of blood pressure on headache in a still unknown manner.

## 1. Introduction

Headache is one of the most common health problems in children and adolescents worldwide [1,2], and a physiologically relevant link between headache and blood pressure has long been suspected [3]. Whereas numerous studies considered arterial hypertension as a possible cause leading to headache [4,5,6,7], other reports did not find a relation between blood pressure and headache [8,9,10]. One important study in adults observed that high systolic and diastolic blood pressure at baseline was associated with a reduced risk of having non-migrainous headache at follow-up 11 years later [11]. Likewise, data from the large, population-based Young-Nord-Trøndelag Health Study in 5847 Norwegian adolescents showed that increasing blood pressure was related to a reduced prevalence of both tension-type recurrent headache and recurrent migraine [12]. Previously, we demonstrated a relationship between elevated blood pressure and both fewer mental health problems and a higher health-related quality of life in German children and adolescents [13]. Based on these observations, we now aimed to assess the hypothesis that headache is inversely related to blood pressure, independent of mental problems and quality of life.

## 2. Methods

### 2.1. Study Population and Data Collection

This post hoc analysis is based on data from the public use file of the German Health Interview and Examination Survey for Children and Adolescents (KiGGS). The KiGGS study was a national survey conducted by the Robert Koch Institute (Berlin, Germany), between 2003 and 2006 following a one-year pretest that collected data to provide information about the mental and physical health of children and adolescents in Germany [14]. The data from the clustered randomly selected sample included sociodemographic characteristics, family economic background, the children’s medical histories and health status, as well as medication use and health-related behaviors. In the first step of the two-stage probability sampling strategy used in the KiGGS study, a sample of 167 municipalities was drawn that were representative of the municipality sizes and the population structure in Germany. In the second step, a random sampling from local population registries in proportion to the age and sex structure of the nationwide German child population was performed [15]. 

The KiGGS baseline survey had a 66.6% response rate, producing a total cohort of 17,641 study participants aged between 0 and 17 years (8656 girls and 8985 boys) [16,17]. A weighting factor was calculated from the total study cohort to correct deviations from the normal distribution for sex, age, nationality, region, and parents’ education level to allow population-based statements. This post hoc analysis focuses on age groups between 11 and 17 years (*n* = 5221) since, for these study participants, information on blood pressure as well as data from both self- and parent-rated psychosocial assessments were available. Using a standard interview question, study participants were asked whether they had experienced none, one, or more than one episode of headache within the last three months. The sample was categorized based on the presence of recurrent headache into those who reported suffering from headache (*n* = 2238) and those who did not (*n* = 2983) [18]. 

All accompanying parents, and the adolescents themselves if over 14 years of age, provided their written informed consent to participate in the survey prior to each interview and examination. The study protocol was approved by the Institutional Review Board of the Charité Universitätsmedizin Berlin as well as the German Federal Data Protection Office.

### 2.2. Clinical Assessments

The local study teams at each sample point, which consisted of five trained members led by a physician experienced in pediatrics, performed clinical interviews and physical examinations, and took blood samples for laboratory analyses. From each study participant, systolic and diastolic blood pressure was recorded using a two-time oscillometric determination with an automatic sphygmomanometer (Datascope Accutorr Plus, Mahwah Township, New Jersey, USA), according to the manufacturer’s algorithm [19]. The two measurements were recorded at rest: the first recording at 5 minutes after sitting down and the second recording at least 2 minutes after a non-stressful physical examination, which was an eye test. Both measurements were performed on the right unclothed arm with the elbow positioned at heart level, unless there were injuries or obstacles such as plaster casts. The blood pressure cuffs covered at least two-thirds of the upper arm, ranging from the axilla to the elbow fold. An influence of the examiner could be excluded as the measurement was performed automatically. For the calculation of the body mass index (BMI), the weight given in kilograms of each participant was divided by the square of the height given in meters.

### 2.3. Laboratory Measurements

For laboratory measurements, blood samples were taken with evacuated EDTA and gel tubes after venipuncture. The samples were refrigerated at 4 °C and transported to a university hospital laboratory (Deutsches Herzzentrum Berlin, Berlin, Germany) for central analysis. Commercial tests were performed according to the specifications given by the manufacturers [20]. Magnesium was measured on Hitachi 917 equipment (Roche Diagnostics, Rotkreuz, Switzerland) using the xylidyl blue assay [20]. All analyses were subject to rigorous internal and external quality control according to the guidelines of the German Medical Association for quantitative laboratory measurements [14].

### 2.4. Psychometric Assessment

The Strengths and Difficulties Questionnaire (SDQ) is a widely used psychometric instrument that detects possible psychiatrically relevant issues such as emotional and behavioral problems [21,22]. The instrument is available in a self-rated version as well as a proxy version filled in by the accompanying parents and caregivers, which uses a slightly different wording more suitable for adults [23]. The SDQ questionnaire contains a total of 25 items divided into five subscales: emotional symptoms, hyperactivity/inattention, conduct problems, peer relationship problems, and prosocial behavior. For each item, respondents marked one of three boxes to indicate whether the item was not true (0), somehow true (1), or certainly true [21]. In addition, a total difficulties score was generated by summing up the scores from all subscales, except for prosocial behavior, with a higher score being indicative of more problems. 

To assess health-related quality of life, the German-language Children’s Quality of Life Questionnaire (KINDL-R), available as a self- and parent-rated version, was used [24,25,26]. The KINDL-R questionnaire contains 24 items covering six dimensions: family, friends, self-esteem, physical well-being, emotional well-being, and everyday functioning. The answers are given in five categories (never, seldom, sometimes, often, or always), and a total sum score was calculated, with higher scores indicating better quality of life.

### 2.5. Statistical Analysis

The study cohort was characterized based on the occurrence of headache by means of descriptive statistics. Means and standard deviations were calculated for continuous variables; percentages and standard deviations are given for categorical variables. Group comparisons between probands with and without headache were performed using χ^2^ tests for categorical variables and Student’s *t* and Mann–Whitney tests for continuous measures. Pearson’s correlation coefficients were calculated to establish the associations between systolic blood pressure and self- and parent-rated SDQ and KINDL-R scores. Sampling weights were used to account for unequal sampling probabilities in terms of age, sex, region (Berlin, East and West Germany), and nationality. 

To test whether health-rated quality of life independently predicted the occurrence of headache, logistic regression models were calculated by entering age, sex, body mass index, contraceptive use, mean systolic blood pressure, and serum magnesium levels as confounding variables. Cox and Snell R^2^ were used as indicators for the amount of explained variance in the regression models. Odds ratios (Exp(B)) and results from Wald tests are also reported. Similar models were adjusted to the same confounders by substituting quality of life with either the self- or parent-rated SDQ score. 

Mediation models were computed using the SPSS macro PROCESS v2.10 created by Preacher and Hayes [27] to study the interaction effects of either self- and parent-rated quality of life or mental distress on the relation between systolic blood pressure and headache. In all mediator analyses, the covariates were kept the same as in the respective regression models to keep the results comparable. The results in the mediator models were considered significant when the 95% confidence interval (95% CI) of the indirect effect did not exceed zero. In all tests, a *p* value of <0.05 was considered statistically significant. Statistical analyses were performed with the software Statistical Package for Social Science (SPSS Statistics, version 27) from IBM Deutschland, Ehningen, Germany.

## 3. Results

### 3.1. Characterization of the Total Study Population

**Figure 1:**
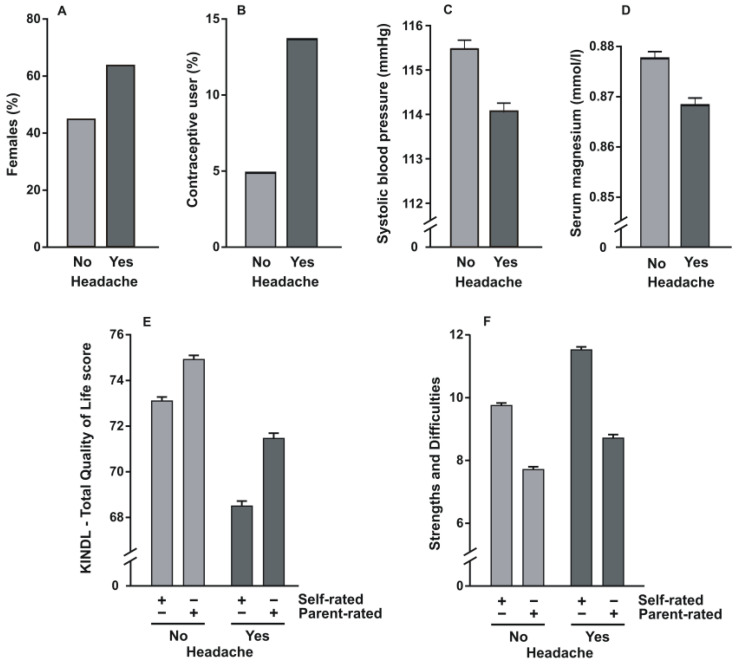
Histograms containing percentages of female study participants (**A**) and contraceptive use (**B**) as well as means and standard errors of systolic blood pressure recordings (**C**), serum magnesium concentrations (**D**), KINDL-R total quality of life (**E**), and Strengths and Difficulties Questionnaire (**F**) scores according to the presence and absence of headache. Symbols (+/−) indicate the respective self- or parent-rated instrument.

In total, 2238 participants from the study cohort experienced frequent headache (42.9%), whereas 2983 did not (Table 1). Study participants with headache were slightly older (15.0 ± 1.9 years vs. 14.7 ± 2.0 years, *p* < 0.001), more frequently female (63.7% vs. 44.9%, *p* < 0.001; Figure 1A), and had a higher body mass index (21.7 ± 4.4 kg/m^2^ vs. 21.0 ± 3.8 kg/m^2^, *p* < 0.001). Oral contraceptive use was more frequently observed in adolescents with versus without headache (13.7% vs. 4.9%, *p* < 0.001; Figure 1B), as was analgesic medication (12.2% vs. 2.8%, *p* < 0.001). Although socioeconomic and migrant status did not significantly differ with respect to headache, systolic blood pressure was slightly and significantly lower in the headache group than in their counterparts who did not report headache (114.0 ± 10.2 mmHg vs. 115.5 ± 11.0 mmHg, *p* < 0.001; Figure 1C). Similarly, serum magnesium levels were significantly lower in participants with headache (0.86 ± 0.06 mmol/L vs. 0.87 ± 0.07 mmol/L, *p* < 0.001; Figure 1D). 

The mean self-rated KINDL-R questionnaire score was statistically lower in the headache group than in those without headache (68.5 ± 10.5 vs. 73.1 ± 9.7, *p* < 0.001; Figure 1E), as was observed for the parent-rated score (71.5 ± 10.5 vs. 75.0 ± 9.9, *p* < 0.001). Similarly, self- and parent-rated SDQ values were both significantly higher in the headache group (11.5 ± 4.6 vs. 9.8 ± 4.4, *p* < 0.001, and 8.7 ± 5.3 vs. 7.7 ± 5.0, *p* < 0.001, respectively; Figure 1F). 

In the total study cohort, systolic blood pressure was positively associated with both self- and parent-rated quality of life (r = 0.030 and *p* = 0.030, and r = 0.036 and *p* = 0.011, respectively) and negatively related to the respective SDQ scores (r = −0.101 and *p* < 0.001, and r = −0.085 and *p* < 0.001, respectively) [13]. A detailed characterization of the total study cohort, including the comparison between participants with and without headache, is given in Table 1.

### 3.2. KINDL-R and SDQ as Predictors for Headache

A series of logistic regression models were computed to confirm whether headache was associated with either health-related quality of life or mental distress, when adjusted for sex, age, body mass index, contraceptive use, blood pressure, and serum magnesium (Table 2). The data showed that both self-rated (Model 1) and parent-rated KINDL scores (Model 2) were significant predictors of headache (self-rated KINDL-R Exp(B) = 0.96, 95% CI = 0.96–0.97, *p* < 0.001 and parent-rated KINDL-R Exp(B) = 0.97, 95% CI = 0.96–0.98, *p* < 0.001). In both models, systolic blood pressure (*p* < 0.001) and magnesium (*p* ≤ 0.039) remained associated with headache. 

Similarly, self-rated (Model 3) and parent-rated SDQ (Model 4) were significant predictors of headache (Exp(B) = 1.08, 95% CI = 1.07–1.10, *p* < 0.001, and Exp(B) = 1.05, 95% CI = 1.04–1.06, *p* < 0.001). In these two models, blood pressure was again associated with headache (*p* < 0.001), whereas magnesium lost its role in predicting the occurrence of headache.

### 3.3. Quality of Life and Distress Mediate the Effect of Blood Pressure on Headache

Mediation models were created to test for possible interactions of either quality of life or mental distress on the relation between systolic blood pressure and headache. Data showed that quality of life was a significant partial mediator of the relation between blood pressure and headache, as the 95% CI for the indirect effect did not include zero. These results were observed for both self- or parent-rated KIND-R scores (Table 3, Models 1 and 2). Similarly, significant indirect effects of systolic blood pressure on the prevalence of headache were also shown for mental distress, as assessed by either self- or proxy-rated SDQ (Table 3, Models 3 and 4). 

## 4. Discussion

Our analyses, based on data from the nationwide, representative German KiGGS study in children and adolescents, showed that the prevalence of headache was inversely related to systolic blood pressure, while it was positively linked to lower quality of life and higher mental distress. The main finding of the present post hoc study is that low blood pressure, reduced quality of life, and high distress are independently associated with the occurrence of recurrent headache. Moreover, psychological parameters such as quality of life and distress seem to mediate the effect of blood pressure on headache, irrespective of whether psychometric data from self- or proxy-rated questionnaires were used in these regression models. 

Several studies have analyzed the relationship between headache and blood pressure and have reported contradictory results. Even though some studies showed a positive relation between hypertension and an increased prevalence of tension-related headaches and migraines [4,5,6,7], it was previously demonstrated that high systolic and diastolic blood pressure are associated with reduction of non-migrainous headache [11,12]. Our data confirm findings from the large, population-based Norwegian Young-Nord-Trøndelag Health Study, demonstrating that blood pressure was inversely related to a reduced prevalence of recurrent episodes of headache. 

The inverse association between headache and blood pressure possibly results from a phenomenon termed hypertension-associated hypalgesia, in which the baroreflex system modulates nociception via endorphinergic and noradrenergic neurons present in the brain and spinal cord [11,12]. In hypertension-associated hypalgesia, an elevated response threshold to noxious stimuli may result in a diminished perception of pain including the occurrence of headache. The antinociceptive state associated with higher resting blood pressure may be due to an attenuated transmission of noxious stimuli in healthy normotensive subjects [28,29,30].

Previously, low systolic and diastolic blood pressure were linked to different psychological disorders such as depression and anxiety in adults [31]. Likewise, we found in data from the KiGGS study that children with elevated blood pressure displayed less distress, had better academic development, and reported higher mental well-being and better quality of life [13]. In addition, it was shown that Korean adults aged ≥19 years with lower blood pressure were more likely to have suicidal thoughts [32]. Pilgrim et al. and others suggested the existence of a hypotensive syndrome characterized by tiredness, dizziness, and headaches, which may include mental symptoms such as anxiety and/or depression [33,34]. Our results are consistent with the findings from these epidemiological studies, suggesting a relationship between low systolic blood pressure and the possibility of a primary psychological disturbance characterized by changes in the quality of life and mental distress. Our models describe low quality of life and high distress (as indicated by SDQ scores) as significant mediators of the relation between low blood pressure and headache. 

Our regression models adjusted for quality of life showed a significant relationship between lower serum magnesium levels and headache problems in German adolescents. The finding of a link between serum magnesium and the prevalence of headache, which was independent of quality of life, is in accordance with data from other studies, showing that juvenile and adult migraineurs had significantly lower serum and red blood cell magnesium concentrations [35,36]. In addition, the percentage of contraceptive users was higher in the cohort with headache, which, however, is not surprising since headache is a common side effect of hormonal contraception for birth control methods [37,38]. In our study cohort, headache was significantly linked to the occurrence of sleep disorders, as nearly one-third of participants with recurrent headache (31.4%) reported symptoms of sleep disorders, whereas this percentage was only 18.6% in the group without headache. When this variable was included as an additional confounder in the regression models, the main finding of this analysis remained unaltered, namely that headache was associated with low systolic blood pressure and overall more psychosocial problems.

Our observations should be interpreted in light of several limitations that are inherent in this post hoc analysis and prohibit any causal interpretation of our findings. The evidence level of our findings is definitively lower compared with randomized controlled trials or with prospectively designed case-control studies, as the survey was not originally designed to assess the specific research question addressed here. Whether blood pressure and the psychological parameters tested have any causal relationship for the development of recurrent headache cannot be conclusively determined from this cross-sectional analysis. Although the large sample size allows for the detection of even weak associations, the correlation coefficients and the effect sizes are very small and, by far, not clinically relevant. 

However, the study also has distinct strengths, which include the large sample size and the method of sampling, which was random selection to ensure nationwide representativeness. The use of validated, clinically accepted assessments in a standardized and blinded manner should also be mentioned as a crucial strength of this analysis. Other strengths result from the clinical interviews and examinations being performed by trained physicians and laboratory measurements conducted under standardized conditions by experienced personnel blinded to the identity of the study participants.

In summary, in this representative, nationwide, and large sample of German adolescents, we demonstrate that headache is inversely associated with systolic blood pressure, and is also linked to lower quality of life and higher mental distress. Psychological parameters may mediate the effect of blood pressure on headache. However, considerably more research is required to confirm these findings and decipher possible physiological mechanisms that may underlie these relationships. 

## Figures and Tables

**Table 1:** Characterization of the weighted German Health Interview and Examination Survey for Children and Adolescents (KiGGS) study cohort aged between 11 and 17 years, including the comparison of different variables in study participants with and without headache.

	Total Study Cohort (*n* = 5221)	Cohort with Headache (*n* = 2238)	Cohort without Headache (*n* = 2983)	*p* Value
Age	14.9 ± 2.0	15.0 ± 1.9	14.7 ± 2.0	<0.001
Sex (males, %)	47.1	36.3	55.1	<0.001
Migrants (%)	17.4	17.3	17.5	0.794
Body mass index (kg/m^2^)	21.3 ± 4.1	21.7 ± 4.4	21.0 ± 3.8	<0.001
Contraceptive use (%)	8.7	13.7	4.9	<0.001
Socioeconomic status (%)	Low: 27.3 Middle: 47.8 High: 25.0	Low: 26.0 Middle: 49.0 High: 25.1	Low: 28.3 Middle: 47.0 High: 24.8	0.157
Systolic blood pressure (mmHg)	114.9 ± 10.7	114.0 ± 10.2	115.5 ± 11.0	<0.001
Magnesium (mmol/L)	0.87 ± 0.07	0.86 ± 0.06	0.87 ± 0.07	<0.001
Self-rated KINDL	70.8 ± 10.1	68.5 ± 10.5	73.1 ± 9.7	<0.001
Parent-rated KINDL	73.1 ± 10.2	71.5 ± 10.5	75.0 ± 9.9	<0.001
Self-rated SDQ score	10.7 ± 4.4	11.5 ± 4.6	9.8 ± 4.4	<0.001
Parent-rated SDQ score	8.2 ± 5.1	8.7 ± 5.3	7.7 ± 5.0	<0.001

Abbreviations: SDQ—Strengths and Difficulties Questionnaire, KINDL—Health Related Quality of Life Questionnaire.

**Table 2:** Results from four binary logistic regression models with headache as the dependent variable adjusted for the following confounders: sex, age, body mass index, contraceptive use, systolic blood pressure, serum magnesium, and mental health.

	**Model 1: self-rated KINDL (R^2^ = 0.088; *p* < 0.001)**			**Model 2: parent-rated KINDL (R^2^ = 0.077; *p* < 0.001)**		
Variable	Exp(B)	95% CI	*P* value	Exp(B)	95% CI	*P* value
Sex	0.619	0.546–0.702	<0.001	0.569	0.502–0.646	<0.001
Age	1.027	0.993–1.063	0.125	1.040	1.005–1.076	0.025
Body mass index (kg/m^2^)	1.032	1.016–1.048	<0.001	1.034	1.018–1.051	<0.001
Contraceptive user (%)	2.083	1.650–2.629	<0.001	2.000	1.588–2.520	<0.001
Blood pressure (mmHg)	0.985	0.979–0.991	<0.001	0.985	0.979–0.991	<0.001
Magnesium (mmol/L)	0.410	0.176–0.956	0.039	0.376	0.162–0.873	0.023
Self-/parent-rated KINDL	0.961	0.955–0.967	<0.001	0.969	0.963–0.975	<0.001
	**Model 3: self-rated SDQ (R^2^ = 0.081; *p* < 0.001)**			**Model 4: parent-rated SDQ (R^2^ = 0.067; *p* < 0.001)**		
Variable	Exp(B)	95% CI	*P* value	Exp(B)	95% CI	*P* value
Sex	0.584	0.516–0.660	<0.001	0.524	0.462–0.595	<0.001
Age	1.057	1.022–1.093	0.001	1.065	1.029–1.102	<0.001
Body mass index (kg/m^2^)	1.030	1.014–1.047	<0.001	1.031	1.015–1.048	<0.001
Contraceptive user (%)	1.965	1.563–2.470	<0.001	1.960	1.559–2.463	<0.001
Blood pressure (mmHg)	0.987	0.981–0.993	<0.001	0.986	0.980–0.992	<0.001
Magnesium (mmol/L)	0.515	0.223–1.187	0.119	0.464	0.201–1.072	0.072
Self-/parent-rated SDQ	1.081	1.067–1.095	<0.001	1.048	1.036–1.061	<0.001

Abbreviations: SDQ—Strengths and Difficulties Questionnaire, KINDL—health-related Quality of Life Questionnaire.

**Table 3:** Mediation models to test for possible interactions of quality of life on the relation between systolic blood pressure and headache, demonstrating the bootstrapped estimates, 95% confidence intervals (CIs), and the amount of variance explained for the direct and indirect effects. Mental distress was measured by the Strengths and Difficulties Questionnaire (SDQ), whereas quality of life was obtained by the KINDL-R questionnaire. Indirect effects of systolic blood pressure on headache (E) are shown for the indicated parameters.

	**Model 1: self-rated KINDL (R^2^ = 0.0454; *p* < 0.001)**			**Model 2: parent-rated KINDL (R^2^ = 0.0119; *p* < 0.001)**		
Variable	Coeff.	95% CI	*P* value	Coeff.	95% CI	*P* value
Age	0.0381	0.0019–0.0743	0.0393	0.0515	0.0151–0.0879	0.0055
Body mass index (kg/m^2^)	0.0241	0.0069–0.0413	0.0060	0.0279	0.0105–0.0452	0.0016
Contraceptive user (%)	0.7392	0.4819–0.9964	<0.001	0.7176	0.4615–0.9738	<0.001
Blood pressure (mmHg)	−0.0145	−0.0213 to –0076	<0.001	−0.0159	−0.0227 to –0.0090	<0.001
Magnesium (mmol/L)	−0.8217	−1.7165–0.0732	0.0719	−0.9651	−1.8606 to –0.0697	0.0346
Quality of life (KINDL-R)	−0.0393	−0.0457 to –0.0328	<0.001	−0.0299	−0.0362 to –0.0236	<0.001
Indirect effect	E = −0.0034, 95% CI = −0.0213 to −0.0076			E = −0.0022, 95% CI = −0.0033 to −0.0012		
	**Model 3: self-rated SDQ (R^2^ = 0.0414; *p* < 0.001)**			**Model 4: parent-rated SDQ (R^2^ = 0.0578; *p* < 0.001)**		
Variable	Coeff.	95% CI	*P* value	Coeff.	95% CI	*P* value
Age	0.0645	0.0288–0.1003	<0.001	0.0708	0.0345–0.1072	<0.001
Body mass index (kg/m^2^)	0.0239	0.0067–0.0411	0.0064	0.0255	0.0082–0.0427	0.0038
Contraceptive user (%)	0.7138	0.4600–0.9676	<0.001	0.7090	0.4549–0.9631	<0.001
Blood pressure (mmHg)	−0.0127	−0.0195 to –0.0059	<0.001	−0.0148	−0.0216 to –0.0080	<0.001
Magnesium (mmol/L)	−0.5696	−1.4566–0.3174	0.2082	−0.7343	−1.6217–0.1531	0.1048
Distress (SDQ)	0.0797	0.0656–0.0938	<0.001	0.0446	0.0322–0.0569	<0.001
Indirect effect	E = −0.0047, 95% CI = −0.0062 to −0.0033			E = −0.0028, 95% CI = −0.0039 to −0.0017		

## Data Availability

Raw data were generated at the Robert Koch Institute. Derived data supporting the findings of this study are available from the corresponding author T.M.
